# Arginase-II activates mTORC1 through myosin-1b in vascular cell senescence and apoptosis

**DOI:** 10.1038/s41419-018-0356-9

**Published:** 2018-02-22

**Authors:** Yi Yu, Yuyan Xiong, Jean-Pierre Montani, Zhihong Yang, Xiu-Fen Ming

**Affiliations:** 1 Cardiovascular and Aging Research, Department of Medicine, Division of Physiology, University of Fribourg, Chemin du Musée 5, 1700 Fribourg, Switzerland; 2 National Center of Competence in Research “Kidney.CH”, Zurich, Switzerland

## Abstract

Type-II L-arginine:ureahydrolase, arginase-II (Arg-II), is shown to activate mechanistic target of rapamycin complex 1 (mTORC1) pathway and contributes to cell senescence and apoptosis. In an attempt to elucidate the underlying mechanism, we identified myosin-1b (Myo1b) as a mediator. Overexpression of Arg-II induces re-distribution of lysosome and mTOR but not of tuberous sclerosis complex (TSC) from perinuclear area to cell periphery, dissociation of TSC from lysosome and activation of mTORC1-ribosomal protein S6 kinase 1 (S6K1) pathway. Silencing Myo1b prevents all these alterations induced by Arg-II. By overexpressing Myo1b or its mutant with point mutation in its pleckstrin homology (PH) domain we further demonstrate that this effect of Myo1b is dependent on its PH domain that is required for Myo1b-lysosome association. Notably, Arg-II promotes association of Myo1b with lysosomes. In addition, we show that in senescent vascular smooth muscle cells with elevated endogenous Arg-II, silencing Myo1b prevents Arg-II-mediated lysosomal positioning, dissociation of TSC from lysosome, mTORC1 activation and cell apoptosis. Taken together, our study demonstrates that Myo1b mediates the effect of Arg-II in activating mTORC1-S6K1 through promoting peripheral lysosomal positioning, that results in spatial separation and thus dissociation of TSC from lysosome, leading to hyperactive mTORC1-S6K1 signaling linking to cellular senescence/apoptosis.

## Introduction

The type II L-arginine:ureahydrolase arginase (Arg-II) is expressed and can be induced in extrahepatic tissues/cells^1–3^. The functions are attributed to hydrolysis of L-arginine to urea and L-ornithine, resulting in decreased cellular L-arginine bioavailability for vascular endothelial nitric oxide synthase (eNOS) to generate the vasoprotective NO, which leads to vascular dysfunction^4–6^. Moreover, recent studies demonstrate that Arg-II is upregulated in human and murine senescent cells and may exert enzymatic activity-independent functions, i.e., non-canonical effects^7–9^. These studies provide evidence that Arg-II causes mitochondrial dysfunction and apoptosis in vascular smooth muscle cells (VSMC) and impairs endothelial autophagy through activation of mechanistic target of rapamycin complex 1 (mTORC1) and ribosomal protein S6 kinase 1 (S6K1) signaling cascade^8,9^, which plays an important role in age-associated vascular dysfunction^7–9^. The underlying molecular mechanisms of Arg-II-induced mTORC1-S6K1 activation remain unknown.

mTOR is a serine/threonine protein kinase that plays an important role in multiple cellular functions through two distinct complexes, i.e., mTORC1 and mTORC2^10^. Deregulation of mTOR signaling is linked to cellular senescence, organism aging and a variety of human pathologies including neurological disease, cancer, diabetes, and cardiovascular disease^10,11^. While the mechanism of mTORC2 activation is less well characterized, intensive studies have elucidated the mechanisms of mTORC1 activation. It has been demonstrated that activation of mTORC1 requires association of mTOR with lysosomes^12^ and dissociation of the inhibitor tuberous sclerosis complex (TSC) from lysosomes^13,14^. Association of mTOR with lysosomes is dependent on the GTPase Rag which allows the mTOR to be close to its activator Rheb (Ras homolog enriched in brain) residing on lysosome surface^12^. Dissociation of TSC from lysosomes can be induced by growth factors and amino acids, resulting in relief of the inhibitory effect of TSC on Rheb and therefore leads to activation of mTORC1 signaling^13,14^. Moreover, lysosomal positioning to cell periphery has also been demonstrated to be essential for mTORC1 activation^15^. However, a link of cell peripheral lysosomal positioning to lysosome-mTOR association and lysosome-TSC dissociation remains unknown.

Evidence has been presented that peripheral positioning of lysosomes is regulated by molecular motor proteins such as the plus-end-directed microtubule-associated molecular motor kinesin superfamily proteins^15–18^ and minus-end-directed dynein^19,20^. Myosin-1b (Myo1b), an unconventional monomeric, non-filamentous class-1 myosin is a protein with actin-associated motor properties and widely expressed in many cells^21^. It consists of an N-terminal motor domain containing the ATP and actin-binding sites, a calmodulin-binding neck region known as an IQ domain, and a C-terminal tail homology domain containing a pleckstrin homology (PH) domain^21^. PH domain enables the direct binding of Myosin tail and phosphatidyl inositol phosphates (PIP, PIP2)^21^. Myo1b was previously shown to predominantly localize to endosomes^22^ and associate with lysosomes^22^. However, a role of Myo1b in lysosomal positioning and mTORC1 activation has not been investigated.

Given the important implications of mTORC1-S6K1 signaling and Arg-II in cellular (dys)functions in numerous diseases and aging, we further elucidated the molecular mechanisms of Arg-II-induced activation of mTORC1-S6K1 signaling pathway. In this study, we first used a hepatocyte cell line lacking endogenous Arg-II expression as a model system to study Arg-II enzymatic activity-independent effect on mTORC1-S6K1 activation and identified Myo1b as a novel mediator of Arg-II-induced activation of mTORC1-S6K1 signaling. This effect of Myo1b is attributed to its PH domain which is required for Myo1b-lysosme association and lysosomal positioning to cell periphery, resulting in spatial separation and thus the dissociation of TSC from lysosomes. This molecular mechanism is further validated in the senescent VSMCs, in which the Arg-II-Myo1b-mTORC1 axis is enhanced, contributing to vascular cell senescence/apoptosis.

## Results

### Arg-II activates mTORC1-S6K1 and induces lysosome re-distribution to cell periphery

**Fig. 1:**
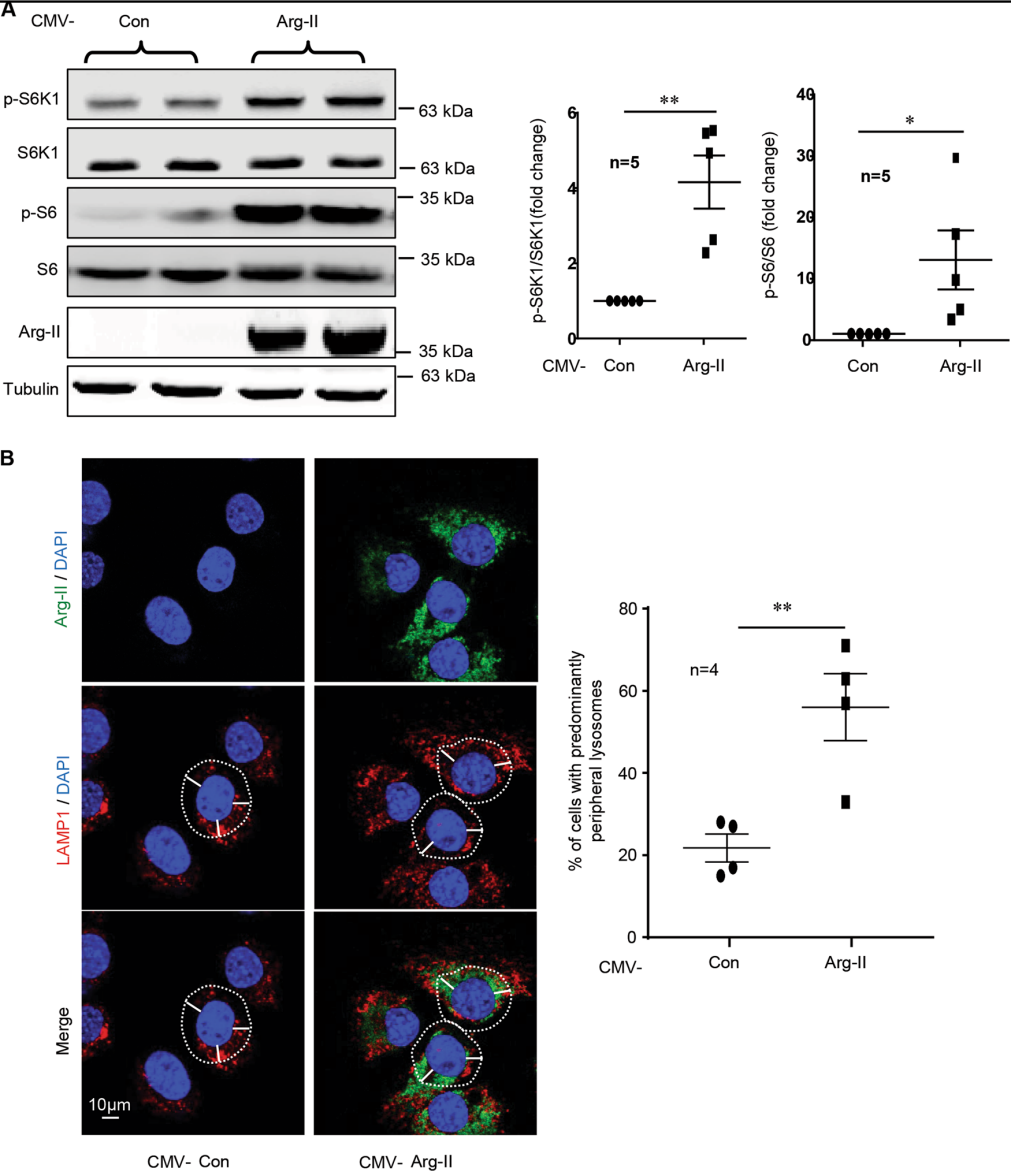
Arg-II induces re-distribution of lysosome to the cell periphery and activates mTORC1-S6K1 pathway. AML12 cells were transduced with rAd/CMV as control (Con) or rAd/CMV-Arg-II (Arg-II). Two days post transduction, cells were serum-starved for 16 h and subjected to (**A**) immunoblotting analysis for S6K1-T389 (p-S6K1), total S6K1, S6-S235/236 (p-S6) and total S6, as well as Arg-II and tubulin. The plot graphs on the right show the quantification of the signals on immunoblots; *p < 0.05; **p < 0.01 vs. Con. (**B**) Immunofluorescence staining of overexpressed Arg-II (green, upper panel) and lysosome marker LAMP1 (red, middle panel) in AML12 followed by counterstaining with DAPI (blue). The merged images are shown in the bottom panel. Quantification of percentage of cells with peripheral lysosmes was presented as plot graphs on the right. Cells, in which more than 50% of LAMP1-positive signals localized in the peripheral region (>10 µm from the nucleus, indicated by white dash line), are defined as cells with predominantly peripheral lysosomes. **p < 0.005 vs. Con. Scale bar = 10 µm.

In the mouse hepatocyte cell line (AML12) which does not express endogenous Arg-II, overexpression of Arg-II stimulated mTORC1-S6K1 signaling as monitored by enhanced S6K1-T389 and S6-S325/326 (Fig. 1A). It is of note that not all cells were transduced to express Arg-II in the culture (Fig. 1B, upper panel). In cells without Arg-II expression, lysosomes as stained with the antibody against a lysosomal marker LAMP1 displayed perinuclear distribution pattern, whereas in cells overexpressing Arg-II, lysosomes were distributed more to the cell periphery (Fig. 1B, middle panel). Confocal microscopy analysis of the co-immunofluorescence staining revealed no co-localization of Arg-II with lysosome (Fig. 1B, bottom panel).

### Arg-II causes TSC dissociation from lysosomes without affecting mTOR-lysosome association

**Fig. 2:**
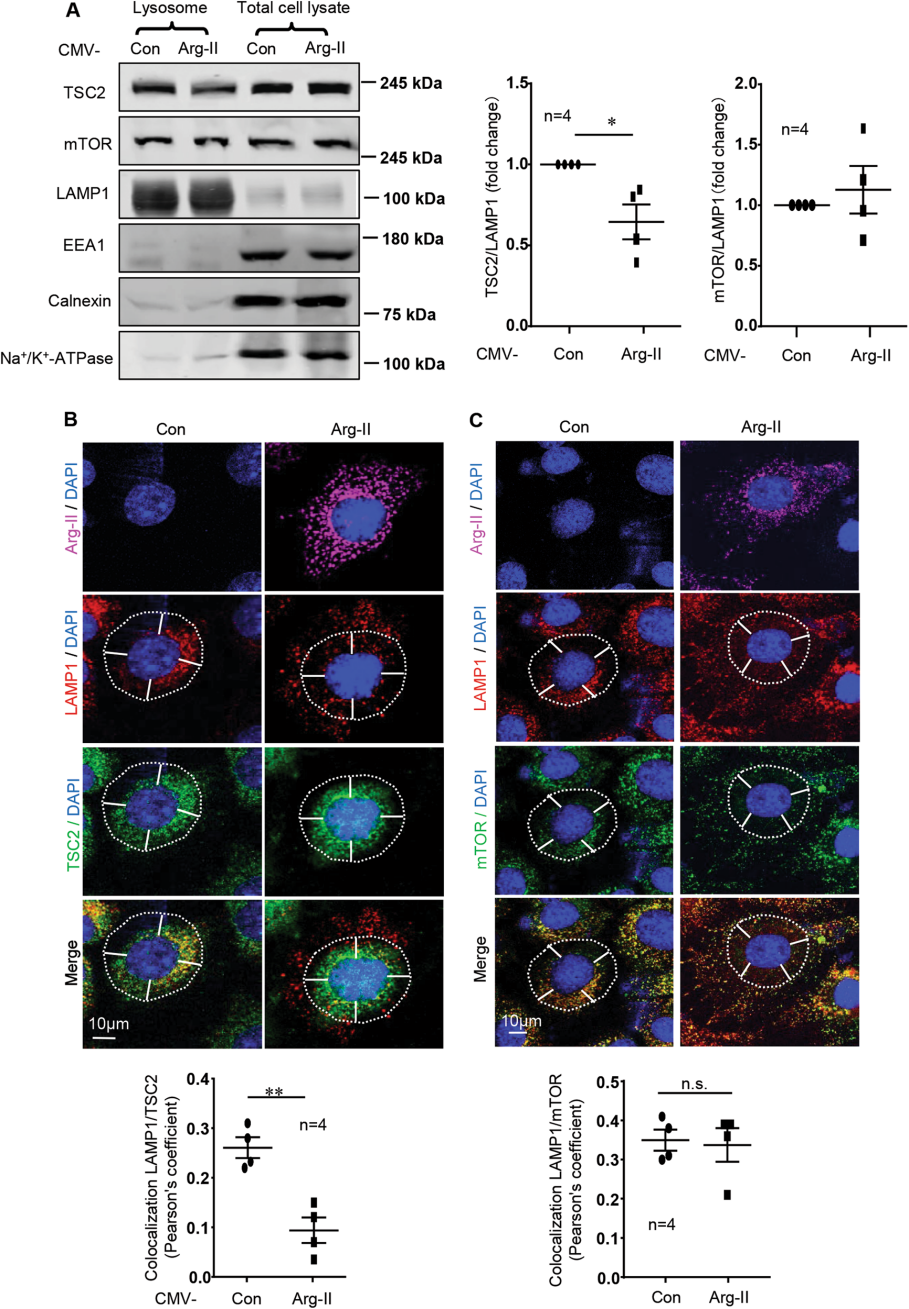
Arg-II causes dissociation of TSC from lysosome without disrupting association of mTOR with lysosomes. AML12 cells were transduced with rAd/CMV as control (Con) or rAd/CMV-Arg-II (Arg-II). 2 days post transduction, cells were serum-starved for 16 h and subjected to (**A**) immunoblotting analysis of TSC2, mTOR, LAMP1 (lysosome marker), EEA1 (marker of early endosomes), Calnexin (marker of endoplasmic reticulum membrane) and Na^+^/K^+^-ATPase (marker of plasma membrane) in lysosome fraction and in total cell lysates. The lysosome fraction was prepared from the total cell lysate. The graphs on the right show the quantification of the signals on immunoblots. Data are presented as mean ± SEM. **(B)** Immunofluorescence staining of Arg-II (pink), LAMP1 (red), and TSC2 (green) followed by counterstaining with DAPI (blue). The merged images for LAMP1, TSC2, and DAPI are also shown (bottom). **(C)** Immunofluorescence staining of Arg-II (pink), LAMP1 (red), and mTOR (green) followed by counterstaining with DAPI (blue); The merged images for LAMP1, mTOR, and DAPI are also shown (bottom). White dashes in images outline boundaries with a predetermined distance of 10 µm from the nucleus, which defines perinuclear (inside the line) and peripheral area (outside the line). Quantification of co-localization of LAMP1 with TSC2 or mTOR is presented as plot graphs below. Scale bar = 10 µm. *p < 0.05 ; **p < 0.01 between the indicated groups. *n.s.* not significant.

We then investigated whether Arg-II affects the association of mTOR and/or TSC with lysosomes. Immunoblotting analysis demonstrated that Arg-II overexpression significantly decreased the lysosomal TSC2 levels and did not affect the total cellular TSC2 (Fig. 2A). In contrast, neither mTOR levels associated with lysosomes nor the total cellular mTOR levels were altered by Arg-II (Fig. 2A). The enrichment of lysosomes was confirmed by markedly increased levels of the lysosome marker LAMP1 and markedly reduced levels of EEA1 (Early Endosome Antigen 1, markers of early endosomes), Calnexin (marker of endoplasmic reticulum membrane) and Na^+^/K^+^-ATPase (marker of plasma membrane) in the lysosomal fraction as compared to the total cell lysates (Fig. 2A). Confocal immunofluorescence staining revealed that in control cells without Arg-II expression, both TSC2 (Fig. 2B) and mTOR (Fig. 2C) were associated with lysosomes at perinuclear area. In cells with Arg-II overexpression, most of TSC2 did not move with lysosomes to cell periphery and retained at perinuclear area, meaning dissociation from lysosomes (Fig. 2B). In contrast, mTOR remained associated with lysosomes and moved together to cell periphery in cells expressing Arg-II (Fig. 2C). To demonstrate that the function of lysosomal peripheral positioning is to dissociate TSC2 from lysosomes, leading to mTORC1 activation, HA-tagged TSC2 (HA-TSC2) was overexpressed in the cells. HA-TSC2 displayed both perinuclear and peripheral distribution (Fig. S1A), is able to associate with peripheral positioned lysosomes without affecting lysosomal peripheral positioning **(**Fig. S1A). Under this condition, activation of mTORC1-S6K1 by Arg-II was inhibited (Fig. S1B), although lysosomal peripheral positioning occurred. These results suggest that Arg-II activates mTORC1-S6K1 pathway by inducing lysosome re-distribution to cell periphery, resulting in spatial separation and thus dissociation of endogenous TSC2 from lysosomes.

### Arg-II activates mTORC1-S6K1 and lysosomal re-distribution and TSC2-lysosome dissociation independently of its enzymatic activity

To investigate whether Arg-II-induced mTORC1-S6K1 activation is dependent on its L-arginine:ureahydrolase activity, recombinant adenoviruses (rAds) expressing various Arg-II truncation mutants with 6His/3HA-tag were constructed. The truncation mutants were designed based on the strictly conserved residues identified from multiple alignments of 31 arginase family enzymes^23^ (Fig. S2A). The mutants were then expressed in the cells **(**Fig. S2B). Interestingly, mutants lacking the C-terminus (i.e., N134, N170, N208, and N244) had no significant effects on mTORC1-S6K1 signaling as assessed by S6K1-T389 and S6-S235/236 levels in the cells, while the C-terminus mutant (C110) which lacks arginase enzymatic activity (Fig. S2), exhibited similar stimulating effect on mTORC1-S6K1 pathway as the full length Arg-II **(**Fig. S2B). The results demonstrate a non-canonical effect of Arg-II on mTORC1-S6K1 activation through its C-terminal domain.

Further experiments showed that the inactive N-terminal mutant HA-N134 did not cause lysosomal peripheral positioning, whereas the inactive C-terminal mutant HA-C110 exerts the same effect as the HA-WT-Arg-II on lysosome cell peripheral positioning and TSC2 dissociation from lysosomes (Fig. S3). This effect of HA-N134, HA-C110, and HA-WT-Arg-II is in accordance with their capability of activating mTORC1-S6K1 pathway as shown in Fig. S2B.

### Identification of Myo1b as a mediator of Arg-II-induced mTORC1-S6K1 activation

**Fig. 3:**
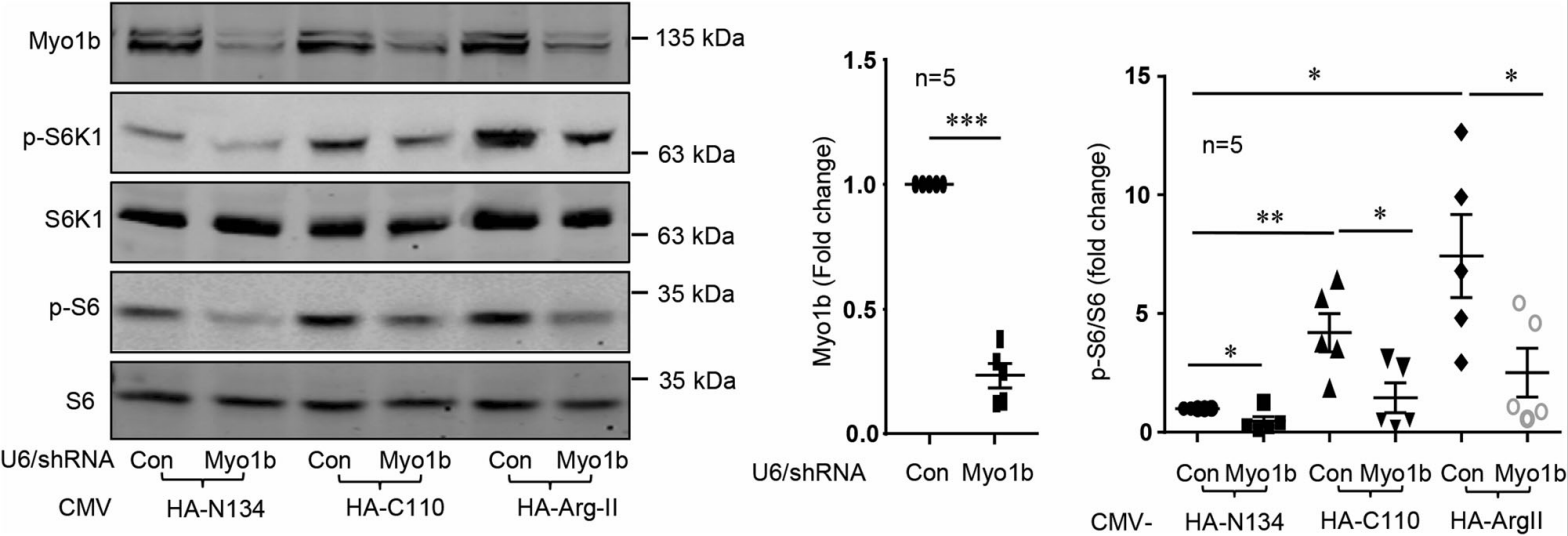
Myo1b mediates non-canonical effects of Arg-II on mTORC1-S6K1 activation. AML12 cells were transduced with rAd/CMV-HA-N134, HA-C110, and HA-Arg-II in the absence or presence of rAd/U6-Myo1b^shRNA^. 2 days post transduction, cells were serum-starved for 16 h. Cell lysates were then prepared and subjected to immunoblotting analysis of Myo1b, S6K1-T389 (p-S6K1), S6K1, S6-S235/236 (p-S6), and S6. The plot graphs on the right show the quantification of the signals on immunoblots. Data are presented as mean ± SEM. *p < 0.05; **p < 0.01; ***p < 0.001 between the indicated groups.

Further experiments were then carried out to identify molecule(s) that possibly mediates the effect of Arg-II in mTORC1-S6K1 activation. Since this effect of Arg-II is attributable to re-distribution of lysosome to cell periphery and Myo1b is a motor protein reported to be associated with lysosome^24^, we examined whether Myo1b could mediate this effect of Arg-II. For this purpose, Myo1b was silenced by adenovirus-mediated expression of shRNA **(**Fig. 3). Silencing Myo1b significantly inhibited the basal as well as the increased level of S6K1-T389 and S6-S235/236 by HA-C110 and HA-WT-Arg-II (Fig. 3), demonstrating a role of Myo1b in the non-canonical effect of Arg-II on mTORC1-S6K1 activation. Of note that no co-localization of Arg-II and Myo1b was observed (Fig. S4).

### Myo1b mediates lysosome re-distribution and TSC-lysosome dissociation by Arg-II

**Fig. 4:**
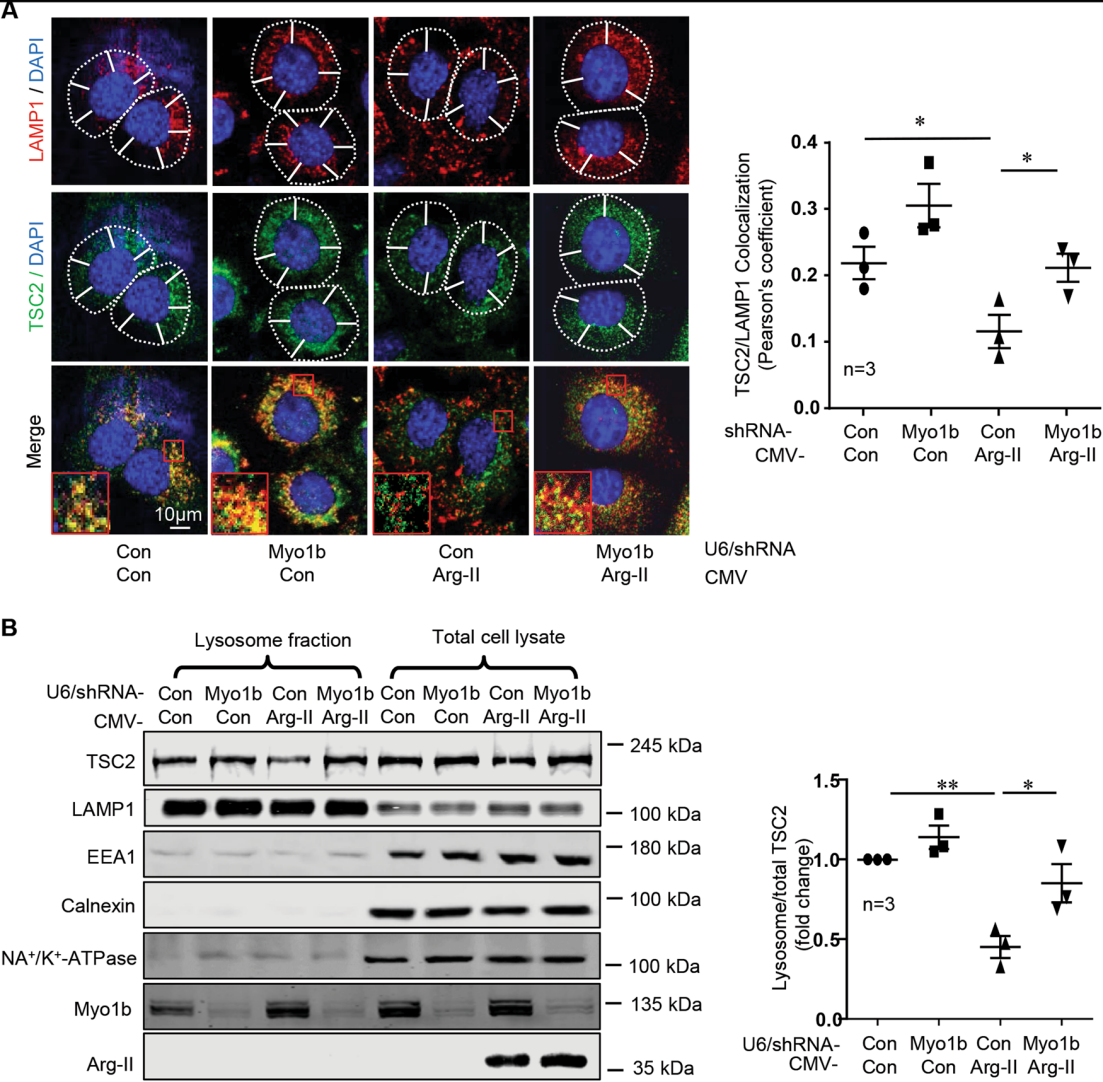
Myo1b is required for Arg-II-induced re-distribution of lysosomes and TSC dissociation from lysosomes. AML12 cells were first transduced either with rAd/U6-LacZ^shRNA^ as control (Con) or rAd/U6-Myo1b^shRNA^ (Myo1b). 18 h post the first transduction, the cells were then transduced either with rAd/CMV as control (Con) or rAd/CMV-Arg-II (Arg-II) for overexpression. Two days post the second transduction, cells were serum-starved for 16 h and subjected to (**A**) immunofluorescence staining for LAMP1 (red) and TSC2 (green) followed by counterstaining with DAPI (blue). The merged images are also shown. Scale bar = 10 µm. White dashes in images outline boundaries with a predetermined distance of 10 µm from the nucleus, which defines perinuclear (inside the line) and peripheral area (outside the line). The plot graphs on the right presents quantification of LAMP1/TSC2 co-localization. **(B)** Immunoblotting analysis of lysosome fraction. The plot graphs on the right presents quantification of lysosomal TSC2/LAMP1 normalized by TSC2/LAMP1 in total cell lysates. Data are presented as mean ± SEM. *p < 0.05; **p < 0.01 between the indicated groups.

Next we determined whether Myo1b is required for Arg-II-induced peripheral positioning of lysosomes and TSC dissociation from lysosomes. In cells, lysosomal positioning to cell periphery and TSC2-lysosome dissociation induced by Arg-II expression were prevented by silencing Myo1b (Fig. 4A). Of note that silencing Myo1b itself tended to enhance TSC2-lysosome association under basal condition, but it did not reach statistical significance (Fig. 4A plot graph). In support of this observation, immunoblotting analysis showed decreased TSC2 levels in lysosomal fraction (but not the total TSC2) from cells expressing Arg-II, which was prevented by Myo1b silencing (Fig. 4B). Myo1b but not Arg-II was detected in lysosome fraction by immunoblotting (Fig. 4B), which supports the observation that there is no co-localization of Arg-II with lysosome by co-immunostaining as shown in Fig. 1B. The results demonstrate that Myo1b mediates Arg-II-induced mTORC1-S6K1 activation by promoting lysosomal re-distribution to cell periphery and TSC2 dissociation from lysosomes.

### Arg-II enhances Myo1b-lysosome association through Myo1b-PH domain leading to mTORC1-S6K1 activation

**Fig. 5:**
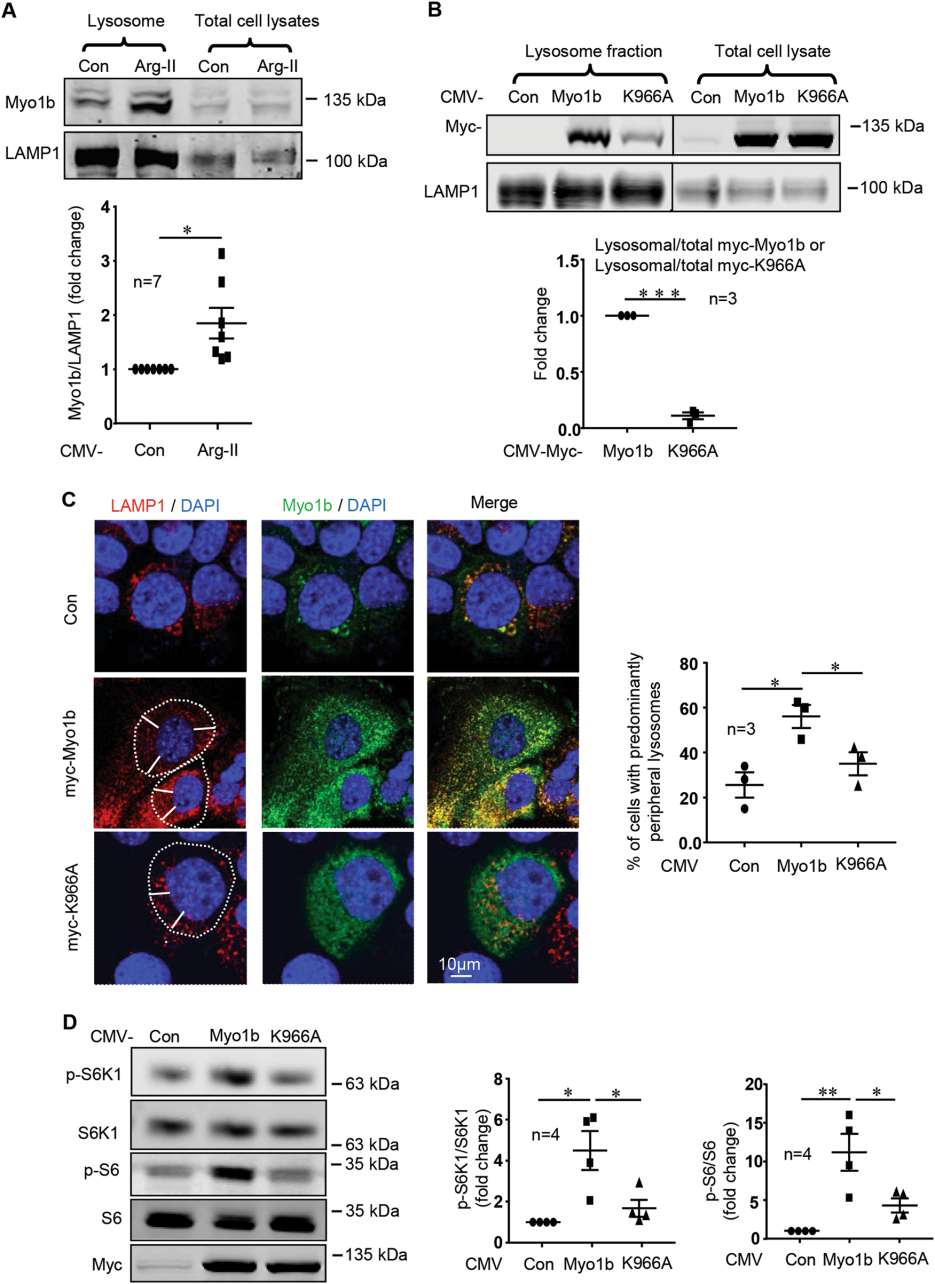
Arg-II promotes association of Myo1b with lysosomes dependently of PH domain of Myo1b, leading to lysosome peripheral positioning. (**A**) Immunoblotting analysis for Myo1b levels in lysosome fraction. AML12 cells were transduced with rAd/CMV as control (Con) or rAd/CMV-Arg-II (Arg-II). **(B**–**D**) AML12 cells were transduced with rAd/CMV as control (Con), or rAd/CMV-myc-Myo1b or the mutant rAd/CMV-myc-K966A for overexpression. Two days post transduction, cells were serum-starved for 16 h and subjected to (**B**) Immunoblotting analysis of myc-Myo1b and myc-K966A in lysosome fractions and total cell lysates using anti-myc antibody. (**D**) Immunofluorescence staining of LAMP1 (red) and myc-Myo1b/myc-K966A (green) followed by counterstaining with DAPI (blue). Scale bar = 10 µm. White dashes in images outline boundaries with a predetermined distance of 10 µm from the nucleus, which defines perinuclear (inside the line) and peripheral area (outside the line). Quantification of cells with predominantly peripheral lysosomes is presented as plot graphs in the right panel. **(D)** Immunoblotting analysis of mTORC1-S6K1 signaling and expression of myc-Myo1b and -K966A. The plot graphs on the right show the quantification of the signals on immunoblots. Data are presented as mean ± SEM. *p < 0.05; **p < 0.01; ***p < 0.001 between the indicated groups.

Since Myo1b was detected in lysosome fraction as shown in Fig. 4B, we determined whether Arg-II induces peripheral lysosome re-distribution through enhancing Myo1b-lysosome association. Overexpression of Arg-II in the cells enhanced Myo1b levels in lysosome fraction without affecting the total Myo1b level (Fig. 5A). To investigate whether the effect of Myo1b is dependent on its PH domain which is believed to mediate its interaction with lysosomes^20,25,26^, myc-tagged WT-Myo1b and a mutant in its C-terminal PH domain (K966A)^26^ were used. As shown in Fig. 5B, large amount of WT-Myo1b but only tiny amount of K966A mutant were detected in the lysosome fraction, although total expression levels of the both were comparable. As compared to the WT-Myo1b, the K966A mutant exhibited markedly reduced capability of inducing lysosome peripheral positioning (Fig. 5C) and TSC2-lysosome dissociation (Fig. S5). In accordance, the K966 A mutant had markedly reduced effect on activating mTORC1-S6K1 (Fig. 5D). These results demonstrate that Arg-II induces lysosomal re-distribution to cell periphery through enhancing association of Myo1b with lysosomes, which is dependent on its PH domain.

### Overexpression of Arg-II activates mTORC1-S6K1 in VSMC involving Myo1b causing VSMC apoptosis

**Fig. 6:**
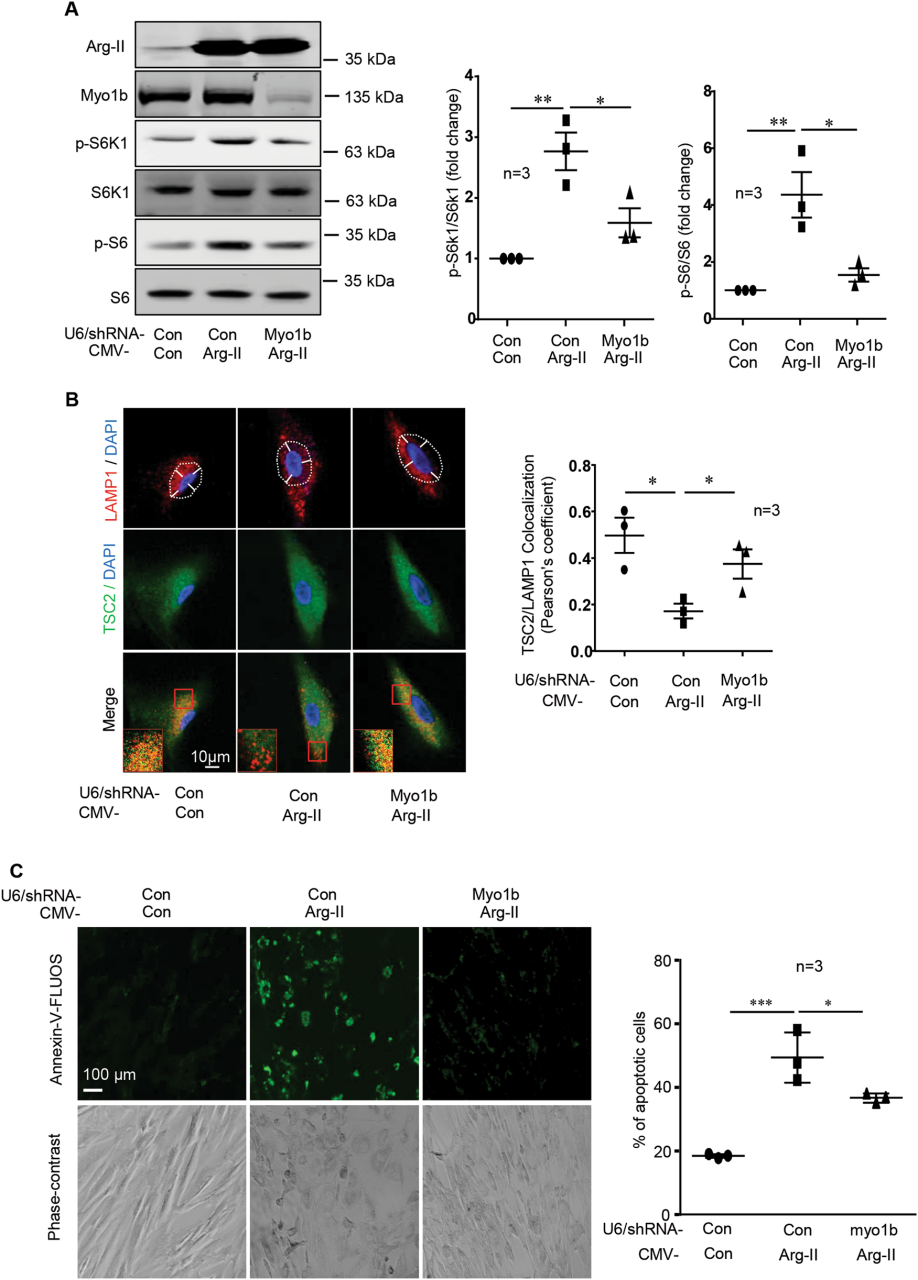
Arg-II induces mTORC1-S6K1 activation in vascular smooth muscle cell (VSMC) via Myo1b. Young human umbilical vein SMCs (HUVSMCs) were first transduced with rAd/U6-LacZ^shRNA^ as control (Con) or rAd/U6-Myo1b^shRNA^, and after 18 h with rAd/CMV as control (Con) or rAd/CMV-Arg-II (Arg-II). After 2 days of the second transduction and 16 h of serum-starvation, the cells were subjected to (**A**) immunoblotting analysis of Arg-II, Myo1b and mTORC1/S6K1 signaling; **(B)** Immunofluorescence staining for LAMP1 (red) and TSC2 (green) followed by counterstaining with DAPI (blue). The merged images are also shown. An enlarged image of the inserts (marked with a red square) of each merged image is shown in the bottom panel. White dashes in images outline boundaries with a predetermined distance of 7.5 µm from the nucleus, which defines perinuclear (inside the line) and peripheral area (outside the line). Of note, HUVSMCs are smaller than AML12 cells. Scale bar = 10 µm. Quantification of TSC2/LAMP1 co-localization is presented in the plot graphs. (**C**) Detection of apoptotic cells by Annexin-V-FLUOS staining (upper panel). The lower panels show phase-contrast images. Plot graphs on the right present quantification of apoptotic cells. Data are presented as mean ± SEM. *p < 0.05; **p < 0.01; ***p < 0.001 between the indicated groups.

To validate the physiological relevance of our findings obtained from AML12 cell model system, we used human VSMC to investigate the role of Arg-II-Myo1b-mTORC1 axis in cell senescence-associated cell apoptoisis. In young VSMC, overexpression of Arg-II is able to activate mTORC1-S6K1 pathway, which was prevented by silencing Myo1b (Fig. 6A). In parallel, overexpression of Arg-II in the young VSMC caused peripheral positioning of lysosomes with concomitant TSC2 dissociation from lysosomes, which was prevented by silencing Myo1b (Fig. 6B). Furthermore, apoptosis induced by Arg-II overexpression in young VSMC as assessed by Annexin-V-FLUOS staining was significantly reduced by Myo1b silencing (Fig. 6C). Phase-contrast images revealed that the cells with Myo1b silencing displayed better morphology as compared with Arg-II-overexpressing cells (Fig. 6C).

### Silencing Myo1b reduces mTORC1-S6K1 and cell apoptosis in senescent VSMC

**Fig. 7:**
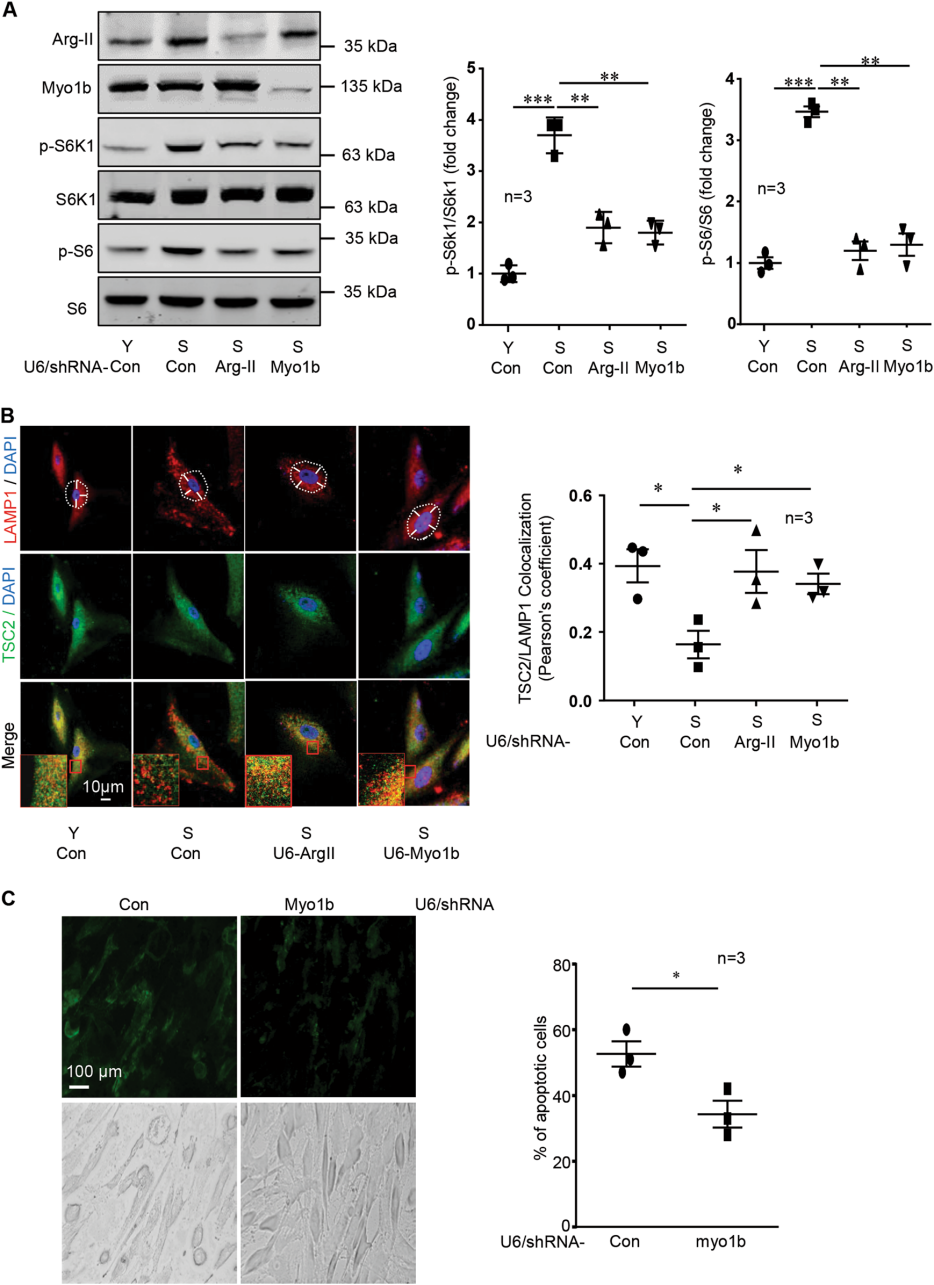
Silencing Myo1b reduces mTORC1-S6K1 and cell apoptosis in senescent VSMC. Young (Y) and senescent (S) VSMC were transduced with rAd/U6-LacZ^shRNA^ as control, rAd/U6-ArgII^shRNA^ or rAd/U6-Myo1b^shRNA^ for silencing. After 2 days of transduction and 16 h of serum-starvation, the cells were subjected to (**A**) immunoblotting analysis of Arg-II, Myo1b, and mTORC1/S6K1 signaling; **(B)** Immunofluorescence staining for LAMP1 (red) and TSC2 (green) followed by counterstaining with DAPI (blue). The merged images are also shown. An enlarged image of the inserts (marked with a red square) of each merged image is shown in the bottom panel. White dashes in images outline boundaries with a predetermined distance of 7.5 µm from the nucleus, which defines perinuclear (inside the line) and peripheral area (outside the line). Scale bar = 10 µm. Quantification of TSC2/LAMP1 co-localization is presented in the plot graphs; **(C)** Detection of apoptotic cells by Annexin-V-FLUOS staining (upper panel). The lower panels show phase-contrast images. Plot graphs on the right present quantification of apoptotic cells. Data are presented as mean ± SEM. *p < 0.05; **p < 0.01; ***p < 0.001 between the indicated groups.

In senescent VSMC, endogenous Arg-II is elevated with concomitant enhanced mTORC1-S6K1 signaling as compared to the young cells (Fig. 7A). Remarkably, the hyperactive mTORC1-S6K1 signaling in senescent VSMC was reduced by silencing either Arg-II or Myo1b (Fig. 7A). The upregulated endogenous Arg-II and hyperactive mTORC1-S6K1 were also accompanied by peripheral lysosome positioning and TSC-lysosome dissociation, which was prevented by silencing either Arg-II or Myo1b in these cells (Fig. 7B). Also apoptosis in senescent VSMC was reduced upon Myo1b silencing (Fig. 7C). These data further support our conclusion that Arg-II-Myo1b-mTORC1-S6K1 signaling is involved in vascular cell senescence/apoptosis. Furthermore, immunoblotting analysis of Myo1b in aortas reveals that protein level of Myo1b is significantly elevated in aortas from aging mice as compared with those of young mice of both males and females (Fig. S6), suggesting a potential role of Myo1b in vascular aging. The age-associated elevation of Myo1b levels are comparable between WT and Arg-II^−/−^ mice (Fig. S6).

## Discussion

Our current study presents the following novel findings: (1) Arg-II exerts a non-canonical effect on mTORC1-S6K1 activation through its C-terminal domain, which is independent of its L-arginine:ureahydrolase activity; (2) This function of Arg-II is mediated through Myo1b by promoting Myo1b association with lysosomes; (3) Myo1b promotes peripheral lysosome positioning, leading to TSC-lysosome dissociation and ultimately mTORC1-S6K1 activation; (4) Myo1b activates mTORC1-S6K1 signaling dependently of its PH domain; (5) Finally, our results demonstrate that Arg-II-Myo1b-mTORC1-S6K1 axis plays a role in senescence-associated VSMC apoptosis.

### Non-canonical effects of Arg-II and Myo1b in activation of mTORC1-S6K1 signaling

Our previous study showed that Arg-II activates mTORC1-S6K1 pathway in VSMC which does not have eNOS expression independently of its enzymatic hydrolase activity^8^. Herein, we verified this non-canonical effect of Arg-II and further characterized that the C-terminal 110 amino acids (C110) fragment of Arg-II lacking any enzymatic activity is critical for this novel function. In contrast, several N-terminal segments are unable to activate mTORC1-S6K1 signaling pathway. Furthermore, we identified Myo1b as a molecule that mediates this effect of Arg-II on mTORC1-S6K1 activation. This conclusion is supported by the fact that silencing Myo1b is able to abolish the ectopic Arg-II-activated mTORC1-S6K1 signaling. Conversely, overexpression of Myo1b in the cells activates mTORC1-S6K1 pathway. The purpose of using the hepatocyte cell line as a model system is that this cell does not express endogenous Arg-II, so that the non-canonical effect of exogenously expressed Arg-II on mTORC1-S6K1 activation can be investigated. It is to note that the results from this model system were verified in human VSMCs. Similar to the hepatocyte model system, the effects of Arg-II overexpression on TSC-lysosome association and mTORC1-S6K1 signaling are reproduced in young VSMCs, which is prevented by silencing Myo1b. The cell apoptosis induced by Arg-II is also inhibited by Myo1b silencing. Importantly, senescent VSMCs express elevated levels of endogenous Arg-II, display reduced TSC2-lysosome association, hyperactive mTORC1-S6K1 signaling, and cell apoptosis. All these senescence-associated functional changes are reduced by silencing either Arg-II or Myo1b, validating the pathophysiological relevance of Arg-II-Myo1b-mTORC1 axis in promoting vascular cell aging.

### Arg-II causes lysosome periphery positioning and TSC2-lysosome dissociation via Myo1b in mTORC1-S6K1 activation

Among other mechanisms, both peripheral lysosomal positioning and dissociation of TSC from lysosomes have been shown to be the independent mechanisms that regulate mTORC1-S6K1 signaling^13–15,27^. However, a link between these two mechanisms has not been proposed. Peripheral lysosomal positioning upon stimulation is believed to bring lysosome-associated mTOR closer to its upstream signaling molecules such as the active form of Akt at the cell membrane^15^ and the peripheral lysosome positioning is reported to be Akt-independent^15^. In contrast, dissociation of TSC from lysosome has been shown to be dependent on Akt which phosphorylates TSC^13^. On the other hand, lysosome recruitment of TSC2 is demonstrated as an universal response to cellular stress that inactivates mTORC1, and presence of any single stress, such as serum starvation or amino acid removal alone, is sufficient to cause TSC2 -lysosome associated in various cell types^27^. Indeed, we show that lysosomes are predominantly positioned perinuclear and associated with TSC2 under the condition of serum-starvation as previously reported^15^. Overexpression of Arg-II or Myo1b alone is capable of promoting peripheral lysosome positioning together with mTOR but not with TSC2. This mechanism results in spatial separation and thus dissociation of TSC2 from lysosome and reliefs the inhibitory effect of TSC2 on the mTOR activator Rheb, leading to activation of mTORC1-S6K1 signaling. Remarkably, when TSC2 is overexpressed, it displays both perinuclear and peripheral distribution, which results in its association also with peripheral lysosomes and thus overrides the effect of Arg-II on mTORC1 activation even when peripheral localization of lysosomes still occurs. These results provide further supporting evidence that lysosome positioning to cell periphery and TSC2-lysosome dissociation, both separately reported by previous studies^13–15,27^, are causally linked as a mechanism for mTORC1 activation. The results demonstrate that peripheral lysosome positioning causes spatial separation of TSC2 from lysosomes, leading to their dissociation. Importantly, this mechanism was validated in senescent VSMC in which elevated endogenous Arg-II causes TSC2-lysosome dissociation and accounts for hyperactive mTORC1-S6K1 signaling.

### Myo1b associates with lysosome, causes lysosome peripheral positioning, and activates mTORC1-S6K1 dependently on its PH domain

We further analyzed detailed molecular basis of Myo1b in activation of Arg-II-mediated mTORC1-S6K1 signaling. Myo1b is a widely expressed unconventional monomeric, non-filamentous class-1 myosin with actin-associated motor properties^21^. In agreement with previous report^24^, we show here that a fraction of Myo1b also associates with lysosomes under control condition. This association is enhanced by Arg-II, which accounts for induction of Myo1b-mediated re-distribution of lysosomes to cell periphery. In support of previous reports that lysosome contains PIP and PIP2^20,25^ and that Myo1b binds specifically with high affinity to PI(4,5)P_2_ and PI(3,4,5)P_3_ via its PH domain^26^, we show here that a Myo1b mutant with point mutation in PH domain (K966A) displays markedly reduced association with lysosomes and capability to activate mTORC1-S6K1 pathway. Remarkably, this mutant loses the capability to induce peripheral lysosomal positioning. These data demonstrate that Myo1b promotes peripheral lysosomal positioning through association with lysosomes, which is dependent on its PH domain. This finding may stimulate the future investigations on the proteins interacting with this domain of Myo1b, which may lead to discovery of new targets of inhibition of mTORC1 signaling and provide therapeutic potential. In an effort to understand how Arg-II promotes Myo1b triggering peripheral positioning of lysosomes, we demonstrate that it is attributable to enhanced association of Myo1b with lysosomes by Arg-II, which is in line with the finding that Myo1b is associated with lysosomes^24^. How Arg-II exactly enhances these associations remains to be investigated.

To date, the well-characterized molecular motor proteins involved in intracellular lysosomal positioning are microtubule-based plus-end-directed kinesins^15–18^ and minus-end directed dynein that are responsible for anterograde and retrograde lysosome movement^19,20^, respectively. Our current study provides evidence that Myo1b is another molecular motor protein in peripheral lysosomal positioning, contributing to mTORC1-S6K1 activation. While Kinesin-like proteins are involved in activation of mTORC1-S6K1 in response to growth factor and nutrient withdrawal/replenishment, Myo1b seems mainly implicated in hyperactive mTORC1-S6K1 signaling linking to vascular aging. This conclusion is supported by the fact that silencing Myo1b not only blunts Arg-II-mediated peripheral lysosomal positioning, dissociation of TSC2 from lysosome and thus hyperactive mTORC1-S6K1 signaling, but also mitigates Arg-II-S6K1-mediated apoptosis in senescent VSMCs. Moreover, a previous study reported enhanced Myo1b mRNA level in aortas of male aging monkey^28^. In current study we observed an elevated protein level of Myo1b in aortas from aging mice of both males and females, suggesting that Myo1b may play a role in vascular aging. It is to note that the age-associated elevation of Myo1b levels in mouse aortas is not affected by Arg-II deficiency, suggesting that the increase in Myo1b protein levels in aging is not regulated by Arg-II. Taking into account that silencing Myo1b selectively attenuates the pathological activation of mTORC1 signaling under the conditions with elevated Arg-II but without significant effects on the cells under basal condition, this selective effect of Myo1b inhibition may provide a new avenue for the development of safer therapies due to its less adversely effects on the normal cells, thus, the off-target effects may be minimal. From all the above mentioned points of view, Myo1b may be a novel target of cardiovascular aging.

## Conclusions

**Fig. 8:**
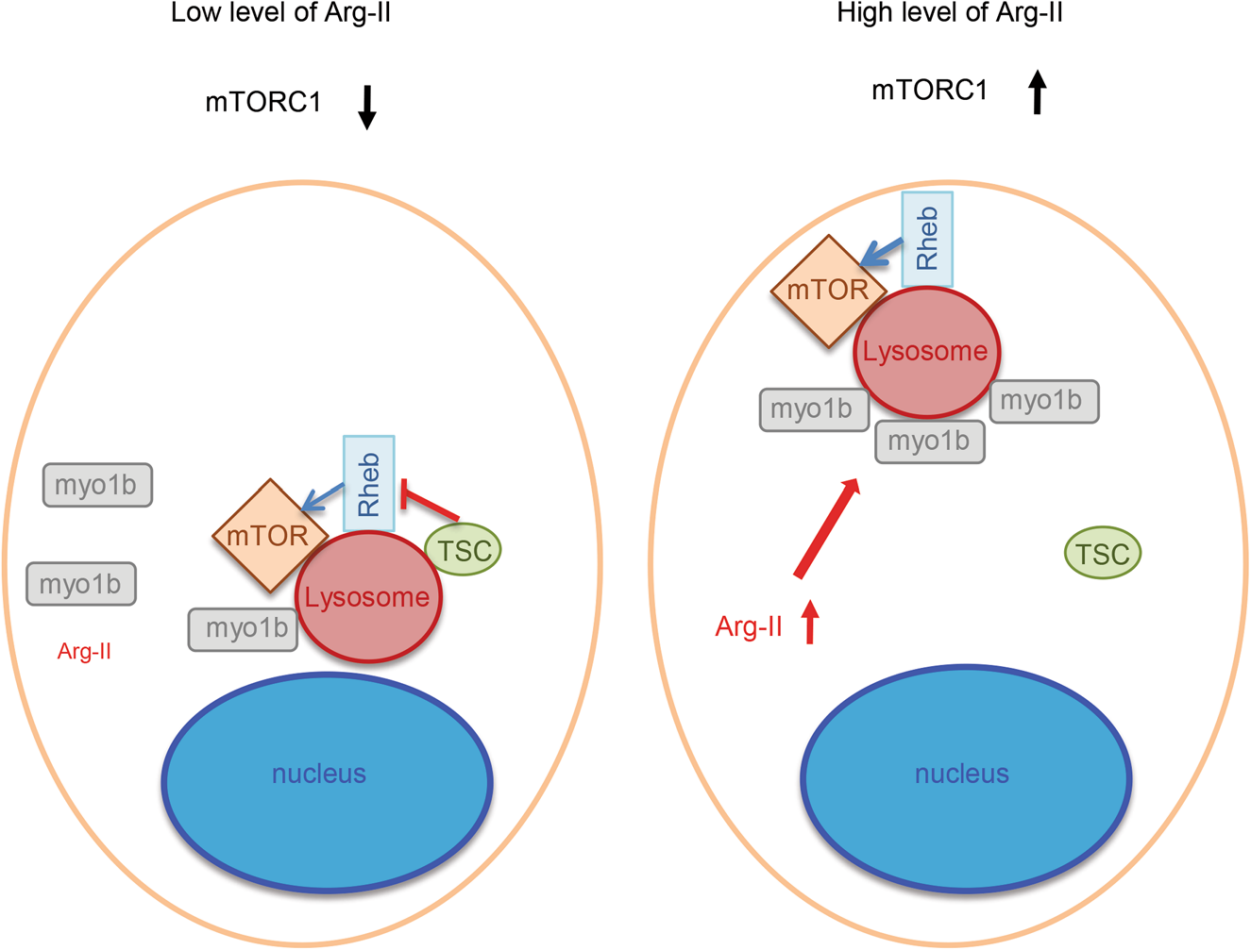
Schematic summary of the major findings of the current study. Arg-II promotes association of Myo1b with lysosomes, which causes peripheral lysosomal positioning, resulting in spatial separation and thus dissociation of TSC from lysosomes. The relief of inhibitory effect of TSC on mTOR upstream activator Rheb ultimately leads to the activation of mTORC1-S6K1 signaling, contributing to vascular cell senescence phenotype, i.e., cell apoptosis, impaired autophagy, and mitochondrial dysfunction

Our findings are summarized in a scheme that integrates our view of how Arg-II activates mTORC1-S6K1 signaling implicated in vascular aging (Fig. 8). Elevated Arg-II in aging, independently of its enzymatic activity, enhances association of Myo1b with lysosome leading to lysosome positioning to cell periphery, which results in spatial separation and dissociation of TSC from lysosome and thus relief of inhibitory effect of TSC on Rheb, and ultimately activation of mTORC1-S6K1 signaling. This represents another novel mechanism that regulates mTORC1-S6K1 pathway and is implicated in vascular aging.

## Materials and methods

### Materials

Reagents were purchased or obtained from the following sources: rabbit (sc-20151) and mouse (sc-393496) antibodies against Arg-II and mouse antibody against Myo1b (sc-393053) were from Santa Cruz Technology Inc (Dallas, USA); mouse antibody against S6 (#2317 s), rabbit antibodies against TSC2 (#4308 P), mTOR (#2983 s), phospho-S6-S235/236 (#2211 s) and phospho-S6K1-T389 (#9234 s) were purchased from Cell Signaling (Danvers, USA); mouse antibody against p70S6K (S6K1, #611260) was from BD Transduction laboratories (New Jersey, USA); rat antibody against LAMP1 (ab24245), rabbit antibody against Myo1b (#194356), rabbit antibody against EEA1 (ab2900) and against calnexin (ab22595), monoclonal mouse antibody against alpha 1 Sodium Potassium ATPase (Na/K-ATPase-α1) [464.6] (ab7671) was from Abcam (Cambridge, UK); mouse antibody against tubulin (T5168) was from Sigma (St. Louis, Missouri, USA). IRDye 800-conjugated affinity purified goat anti-rabbit IgG F(c) was purchased from LI-COR Biosciences (Lincoln, Nebraska USA); goat anti-mouse IgG (H + L) secondary antibody Alexa Fluor® 680 conjugate, goat anti-mouse IgG (H + L) secondary antibody Alexa Fluor® 488 conjugate, goat anti-rabbit IgG (H + L) secondary Antibody Alexa Fluor® 488 conjugate, goat anti-rabbit IgG (H + L) secondary antibody Alexa Fluor® 594 conjugate, goat anti-rat IgG (H + L) secondary Antibody Alexa Fluor® 488 conjugate, goat anti-rat IgG (H + L) secondary antibody Alexa Fluor® 546 conjugate, goat anti-rat IgG (H + L) secondary antibody Alexa Fluor® 680 conjugate were from Invitrogen/Thermo Fisher Scientific (Waltham, MA USA). Insulin-transferrin-selenite sodium and dexamethasone were from Sigma (St. Louis, Missouri, USA). All cell culture media and materials were purchased from Gibco/Thermo Fisher Scientific (Waltham, MA USA).

### Generation of expression vectors

Generation of rAd expressing various deletion mutants of Arg-II driven by cytomegalovirus (CMV) promoter (rAd/CMV-N134, rAd/CMV-N170, rAd/CMV-N208, rAd/CMV-N244, rAd/CMV-C110, rAd/CMV-HA-ArgII) (Fig. S2) was carried out with the Gateway technology (Invitrogen Life Technologies, Carlsbad, California, USA) according to the manufacturer’s instructions. The plasmid pENTR11-6His-3HA-mArgII containing mouse Arg-II cDNA with 6 × His/3 × HA tag at N-terminal was constructed in our laboratory and used as template. Each fragment was amplified by PCR with 6 × His/3 × HA tag at its N-terminal according to the primers below (the underline indicates restriction enzyme sites): N134(1-134aa) forward: 5′-ACGCGTCGAC ATGCACCATCACCATCATCA-3′ (*Sal I*), reverse: 5′-AAGGAAAAAAGCGGCCGCCTAGCGGTGCCGGGCG-3′ (*Not I*); N170(1-170aa) forward: 5′-ACGCGTCGAC ATGCACCATCACCATCATCA-3′(*Sal I*), reverse:5**′-**AAGGAAAAAAGCGGCCGCCTATTCTTTGATGAGAAAGGA-3′ (*Not I*); N208(1-208aa) forward: 5′-ACGCGTCGAC ATGCACCATCACCATCATCA-3′ (*Sal I*), reverse: 5′-AAGGAAAAAAGCGGCCGCCTAAATAAAATGTTCAGGAGG-3′ (*Not I*); N244(1-244aa) forward: 5′-ACGCGTCGAC ATGCACCATCACCATCATCA-3′ (*Sal I*), reverse: 5′-AAGGAAAAAAGCGGCCGCCTACCTCTGCCTTTTGC-3′ (*Not I*); C110(245-354aa) forward: 5′-AGCTTTGTTTAAACCCAATCCACCTGAGTTTTGATATTGA-3′ (*ME I*), reverse: 5′-AAGGAAAAAAGCGGCCGCCTAAATTCTCACACATTC-3′(*Not I*); HA-Arg-II (1-354aa) forward: 5′-ACGCGTCGAC ATGCACCATCACCATCATCA-3′(*Sal I*), reverse: 5′-AAGGAAAAAAGCGGCCGCCTAAATTCTCACACATTC-3′ (*Not I*).

Generation of rAd expressing shRNA targeting mouse and human Myo1b driven by the U6 promoter (rAd/U6- mMyo1b^shRNA^ and rAd/U6- hMyo1b^shRNA^, respectively) was also carried out with the Gateway Technology. The targeting sequences are indicated in boldface below (only the sense strand is shown):

mMyo1b-shRNA:

5′- CACC**GGAGCTCCTCTACAAGCTTAA**CGAATTAAGCTTGTAGAGGAGCTCC -3′

hMyo1b-shRNA:

5′- CACC**GGGCTTTATGGATCATGAAGC**CGAAGCTTCATGATCCATAAAGCCC -3′.

The expression plasmids encoding myc-Myo1b and-K966A were kindly provided by Lynne M. Coluccio^26^. rAd/U6-LacZ^shRNA^, rAd/U6-Arg-II^shRNA^, rAd/CMV empty vector and rAd/CMV-Arg-II were generated as previously described^7^. The expression plasmid encoding HA-TSC2 was a gift from Kunliang Guan (Addgene plasmid # 24939, Cambridge, MA USA).

### Cell culture, adenoviral transduction, and transfection

Culture of young and senescent human umbilical vein smooth muscle cells (HUVSMC) and transduction of HUVSMC by rAd were performed as previously described^8,29^. The alpha mouse liver 12 cell line (AML12) was purchased from ATCC (CRL-2254). AML12 were cultured in petri dishes coated with 1% gelatin and were maintained in DMEM /Nutrient Mixture F-12 Ham supplemented with 10% HIFBS, insulin-transferrin-selenite sodium and dexamethasone as described previously^30^. Cells were transduced with the rAd at titers of ~200 multiplicities of infection and then cultured in complete medium for 2 days and then switched to serum-free medium overnight before experiments. Transfection of the cells was performed using Lipofectmine 3000 Transfection Reagent (L3000008, Invitrogen/ Thermo Fisher Scientific) (Waltham, MA USA) according to manufacturer’s instructions.

### Animals

Arg-II^−/−^ mice were kindly provided by Dr. William O’Brien^31^ and backcrossed to C57BL/6 J for more than eight generations. Wild type (WT) and Arg-II^−/−^ offspring (F2) from hetero/hetero (F1) cross were interbred to obtain WT and Arg-II^−/−^ mice (F3), respectively, for our experiments. The mice were maintained in conventional conditions: 23 °C, 12-h light-dark cycle and fed a normal chow and had free access to tap water. Young (5–7 months) and old (22–24 months) mice were sacrificed as previously described^32^. Aortas was isolated and snap-frozen in liquid nitrogen and kept at −80 °C until processed. Animal work was approved by the Ethical Committee of Veterinary Office of Fribourg (Nr. 2013_08_FR), Switzerland and was performed in compliance with guidelines on animal experimentation at our institution.

### Immunoblotting

Cell and aorta lysate preparation, sodium dodecyl sulfate polyacrylamide gel electrophoresis, transfer of sodium dodecyl sulfate gels to an Immobilon-P membranes (Millipore) were performed as previously described^7^. The resultant membrane was first incubated with the corresponding primary antibody at 4 °C overnight with gentle agitation after blocking with 5% skimmed milk. The blot was then further incubated with a corresponding anti-mouse (Alexa fluor 680 conjugated) or anti-rabbit (IRDye 800 conjugated) or anti-rat (Alexa fluor 680 conjugated) secondary antibody. Signals were visualized using Odyssey Infrared Imaging System (LI-COR Biosciences, Lincoln, Nebraska USA). Quantification of the signals was performed using NIH Image 1.62 software (U. S. National Institutes of Health).

### Immunofluorescence staining

Cells grown on glass coverslips were fixed with 4% paraformaldehyde, permeabilized with 0.2% Triton X-100, and blocked with 1% BSA in PBS. Coverslips were incubated with primary antibody overnight at 4 °C, followed by incubation with Alexa Fluor-labeled secondary antibodies for 1 h at room temperature, and mounted. Images were acquired through 63 × objectives with Leica TCS SP5 confocal laser microscope. All of the confocal images shown in the current study correspond to representative images of one confocal section throughout the focal plane of the nucleus of one cell taken at the same exposure and magnification.

### Quantification of lysosomal distribution

Quantification of lysosomal distribution was performed according to the description with modification from Korolchuk et al^15^. In brief, cells were categorized into perinuclear-dominant lysosomal distribution (more than 50% of LAMP1-positive signals localized in the perinuclear region, < 10 µm (AML12 cells) or 7.5 µm (HUVSMCs) from the nucleus) and peripheral-dominant distribution (more than 50% of LAMP1-positive signals localized in the peripheral region, >10 µm (AML12 cells) or 7.5 µm (HUVSMCs) from the nucleus). Of note, HUVSMCs are smaller than AML12 cells. Quantification is based on at least three independent experiments, each carried out in duplicate, and around 150 cells with only one nucleus were counted in each slide; the quantification was blinded calculated. The data are presented as proportion of cells with predominantly (>50%) peripheral lysosomes.

### Quantification of TSC2/lysosome co-localization

Co-localization of TSC2/lysosome in confocal microscopy experiments was quantified using the Coloc2 plugin of Fiji software based on at least three independent experiments. The Pearson’s co-localization coefficient of TSC2/LAMP1 (with threshold) was used to quantify the co-localization of TSC2 and lysosomes. For each condition, 20–30 cells were examined.

### Lysosome isolation

The Lysosomes of transduced AML12 cells were isolated according to the protocol of Lysosome Enrichment Kit for Tissues and Cultured Cells (Invitrogen, # 89839).

### Detection of apoptotic cells

Apoptosis of transduced HUVSMCs was detected with Annexin-V-FLUOS Staining Kit (Roche Applied Science #1988549, Basel, Switzerland) according to the manufacturer’s instructions. Quantification was presented by the ratio of apoptotic cells/total cells.

### Statistics

The Kolmogorov–Smirnov test was used to first determine whether the data deviate from Gaussian distributions. For normal distributed values, statistical analysis was performed with the Student’s *t*-test for unpaired observations or analysis of variance with Bonferroni’s post-test, and data are given as means ± SEM. For non-normal distributed values, non-parametric statistical analysis was performed with the Kruskal–Wallis test with a Dunn’s multiple comparison post-test, and data are expressed as medians with interquartile range. *p* ≤ 0.05 is considered to indicate a statistical difference. The *n* indicates the number of individual animals used or of individual experiments when conducted with cells.

## Electronic supplementary material

Supplemental Figures
