# Supplementary material for: Arginase-II activates mTORC1 through myosin-1b in vascular cell senescence and apoptosis
Source: Cell Death Dis. 2018 Feb 22;9(3):313. doi: 10.1038/s41419-018-0356-9 (PMC5833809; doi:10.1038/s41419-018-0356-9)
Supplement: Supplementary file 1 — Supplemental Figures [file 41419_2018_356_MOESM1_ESM.pdf]

Supplementary Information

Supplementary Information for:

Arginase-II activates mTORC1 through myosin-1b
in vascular cell senescence and apoptosis

Yi Yu[1#], Yuyan Xiong[1#], Jean-Pierre Montani[1,2], Zhihong Yang[1,2]*, and Xiu-Fen Ming[1,2]*

[1]Cardiovascular and Aging Research, Department of Medicine, Division of Physiology, University of Fribourg, Chemin du Musée 5, 1700 Fribourg, Switzerland
[2]National Center of Competence in Research "Kidney.CH", Switzerland

*Corresponding author
[#] These authors contributed equally to this work.

Running title: Arginase-II activates mTORC1 through myosin-1b

Address for correspondence:
Zhihong Yang, MD or
Xiu-Fen Ming, MD, PhD
Cardiovascular and Aging Research
Department of Medicine
Division of Physiology
University of Fribourg
Chemin du Musée 5
CH-1700, Fribourg
Switzerland
Tel: 0041-26-300 85 93
Fax: 0041-26-300 97 34
Email: zhihong.yang@unifr.ch
or xiu-fen.ming@unifr.ch

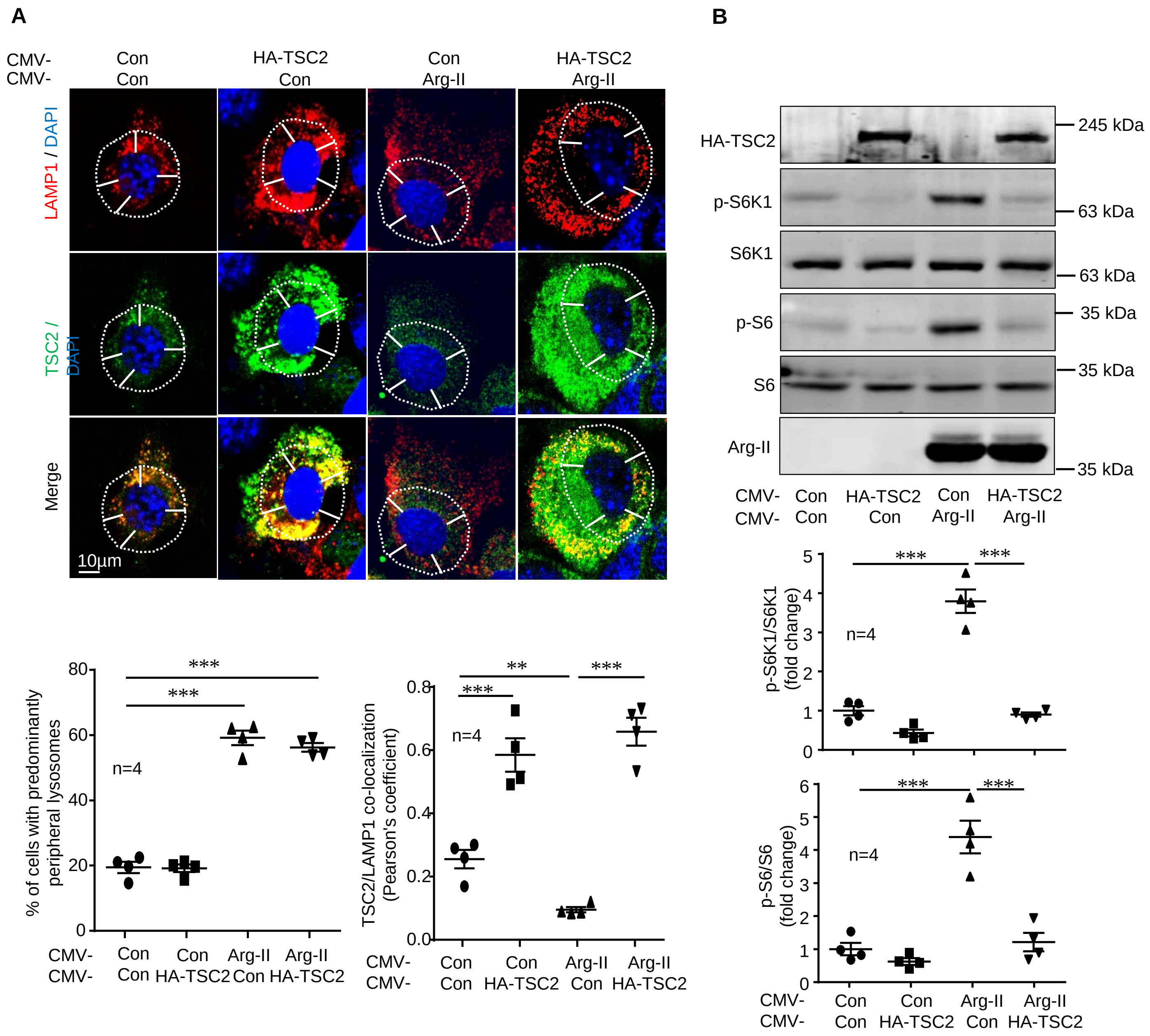

Fig. S1. Overexpressed TSC2 displays both perinuclear and peripheral distribution, which results in its association also with peripheral lysosomes and thus overrides the effect of Arg-II on mTORC1 activation even when peripheral localization still occurs. AML12 cells were transfected with pcDNA3 vectors as control (Con), HA-TSC2, -Arg-II or -Arg-II plus HA-TSC2 as indicated. Two days post transfection, cells were serum-starved for 16 hours and subjected to **(A)** Immunofluorescence staining for lysosome marker LAMP1 (red) and TSC2 (green) followed by counterstaining with DAPI (blue). White dash line in images outline boundaries with a predetermined 10 μm of distance from the nucleus which defines perinuclear (inside the line) and peripheral area (outside the line). Scale bar =10 μm. The plot graphs below show the quantifications of fluorescence signals. Cells, in which more than 50% of LAMP1-positive signals localized in the peripheral area, are defined as cells with predominantly peripheral lysosomes. **(B)** Immunoblotting analysis for HA-TSC2, S6K1-T389 (p-S6K1), total S6K1, S6-S235/236 (p-S6), total S6 and Arg-II. The graphs below show the quantification of the signals on immunoblots. $^{**}p<0.01$, $^{***}p<0.001$ between indicated groups.

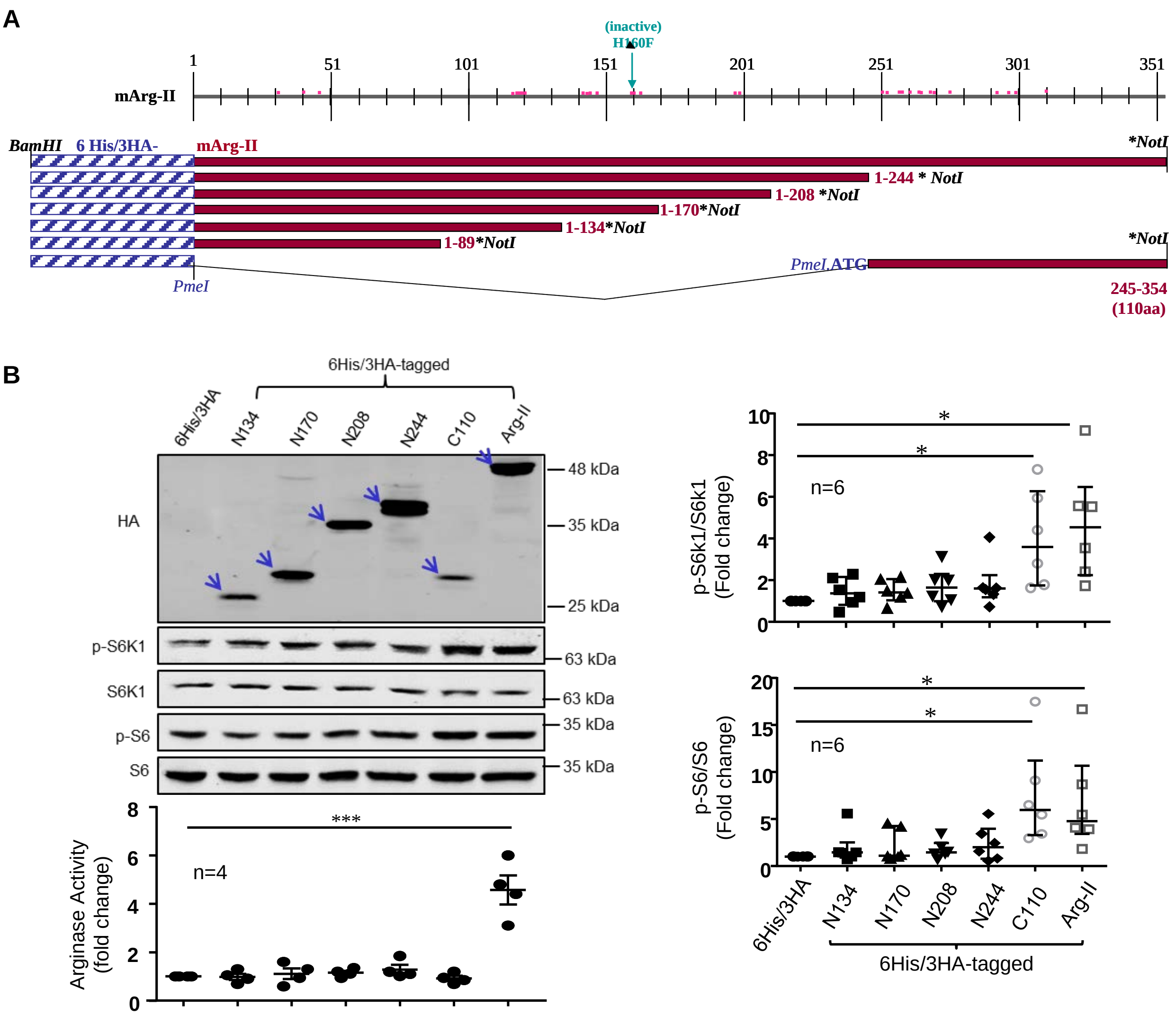

Fig. S2. Arg-II mutants and their effect on mTORC1-S6K1 signaling. (A) Schematic presentation of strictly conserved residues among 31 arginase family enzymes and the different mouse Arg-II (mArg-II) truncation mutants. The upper black line represents the full length of mArg-II protein (354 amino acids, 354 aa) with indicated numbering of the aa. The pink points on the black line indicate the strict conserved residues identified from multiple alignment of 31 arginase family enzymes. Below are the different 6His/3HA-tagged mArg-II truncation mutants referred to as N134 (1-134 aa), N170 (1-170 aa), N208 (1-208 aa), N244 (1-244 aa) and C110 (245-354 aa). **(B)** Effect of various truncation mutants on Arg-II-induced activation of mTORC1-S6K1 signaling. Mouse hepatocytes (AML12) were transduced with recombinant adenovirus harboring various 6His/3HA-tagged truncation mutants of mArg-II driven by CMV promoter. Lysates were prepared after serum-starvation for 16 h on day 2 post transduction and subjected to arginase activity and immunoblotting analysis of Arg-II mutants using anti-HA antibody (blue arrows indicate the corresponding HA-tagged Arg-II mutants), S6K1-T389 (p-S6K1), total S6K1, S6-S235/236 (p-S6), and total S6. The bar graphs on the right show quantification of p-S6K1/S6K1 and p-S6/S6. The bar graph below shows arginase activity. *p<0.05 between indicated groups. ***p<0.001 between indicated groups.

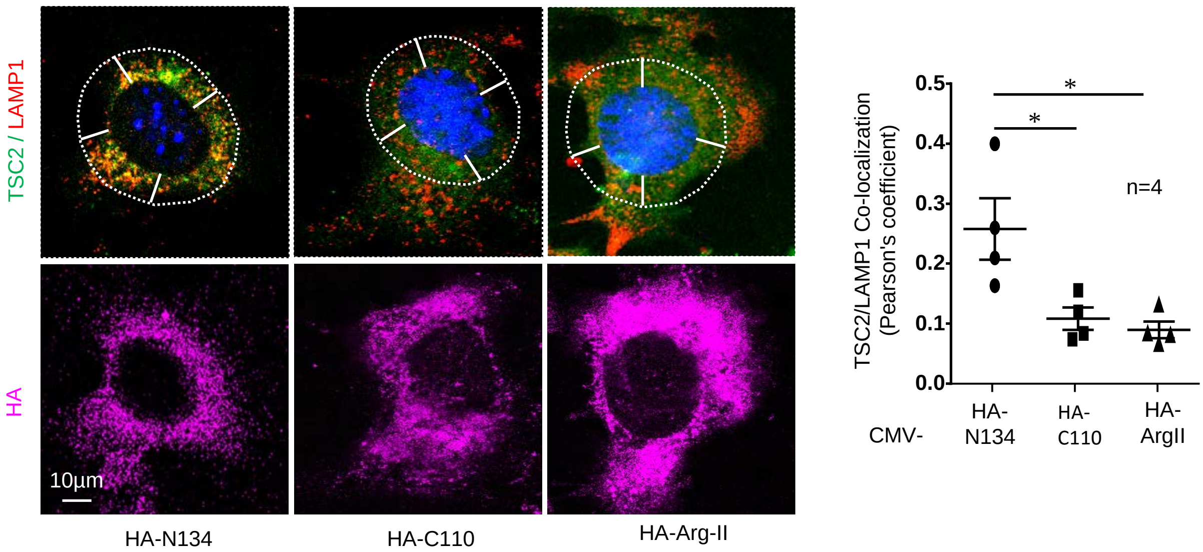

Fig. S3. Arg-II causes lysosome re-distribution and TSC2-lysosome dissociation independently of its enzymatic activity. AML12 cells were transduced with rAd/CMV-HA-N134, -C110 and -full-length Arg-II (HA-N134, HA-C110, HA-Arg-II, respectively). Two days post transduction, cells were serum-starved for 16 hours and subjected to immunofluorescence staining of TSC2 (green), LAMP1 (red) and HA-tagged Arg-II mutants (pink). White dashes in images outline boundaries with a predetermined distance of 10 µm from the nucleus, which defines perinuclear (inside the line) and peripheral area (outside the line). The plot graph on the right presents quantification of TSC2/LAMP1 co-localization. Data are presented as mean ± SEM. *$p<0.05$ between the indicated groups.

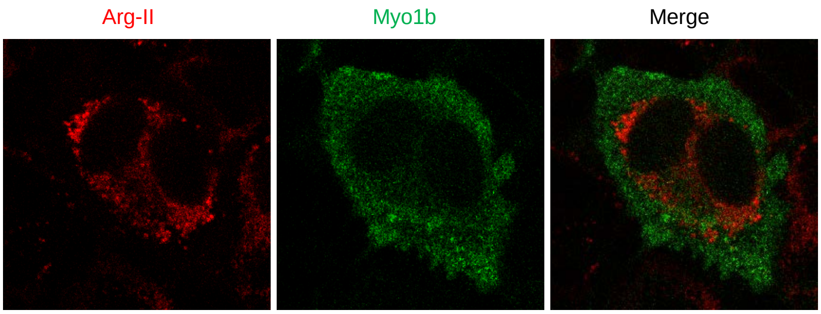

Fig. S4. No co-localization of Arg-II and Myo1b. Co-immunostaining of Arg-II (red) and Myo1b (green) in AML12 cells transduced with rAd/CMV-Arg-II. The merged image is shown.

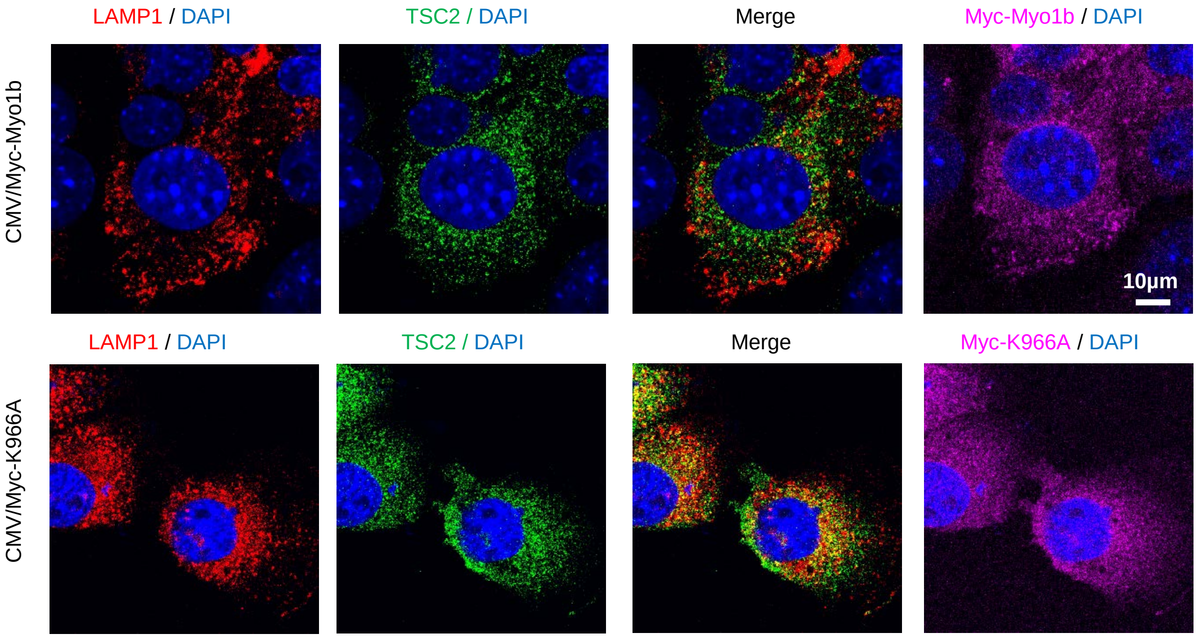

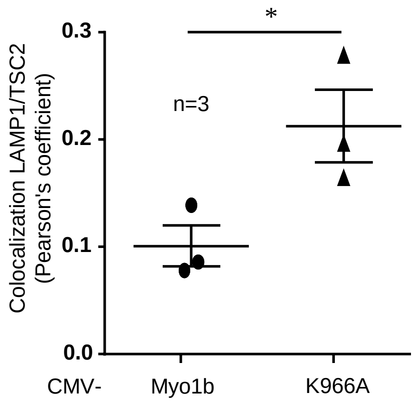

Fig. S5. Myosin 1b but not its mutant in PH domain induces dissociation of TSC from lysosomes. AML12 cells were transduced with rAd/CMV-Myc-Myo1b or rAd/CMV-Myc-K966A mutant in PH domain. Two days post transduction, cells were serum-starved for 16 hours and subjected to immunostaining for LAMP1 (red), TSC2 (green) and Myc-Myo1b and -K966A (pink) followed by counterstaining with DAPI (blue). The merged images for LAMP1, TSC2 and DAPI are also shown. Quantification of TSC2/LAMP1 co-localization is presented as plot graphs below. *$p<0.05$ vs myc-Myo1b.

A

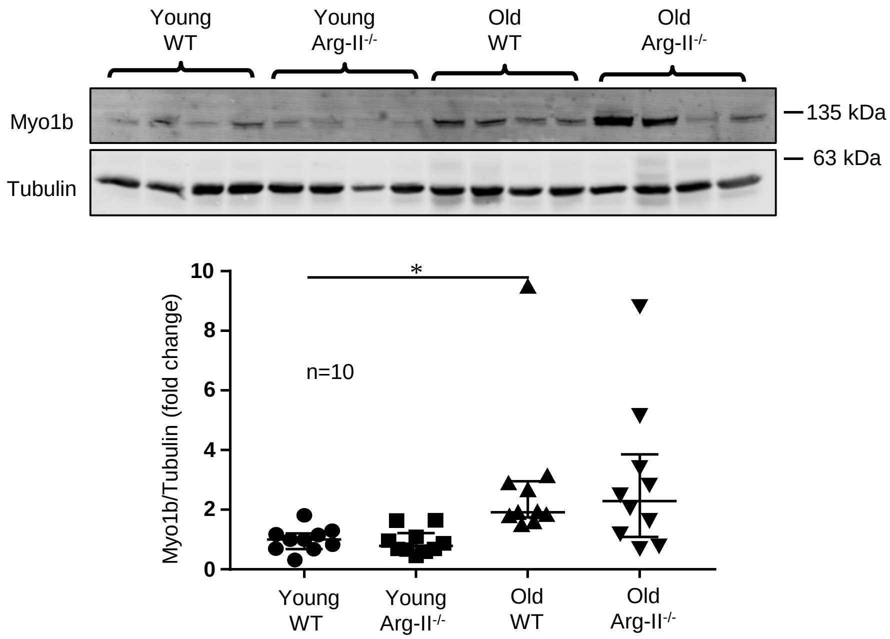

B

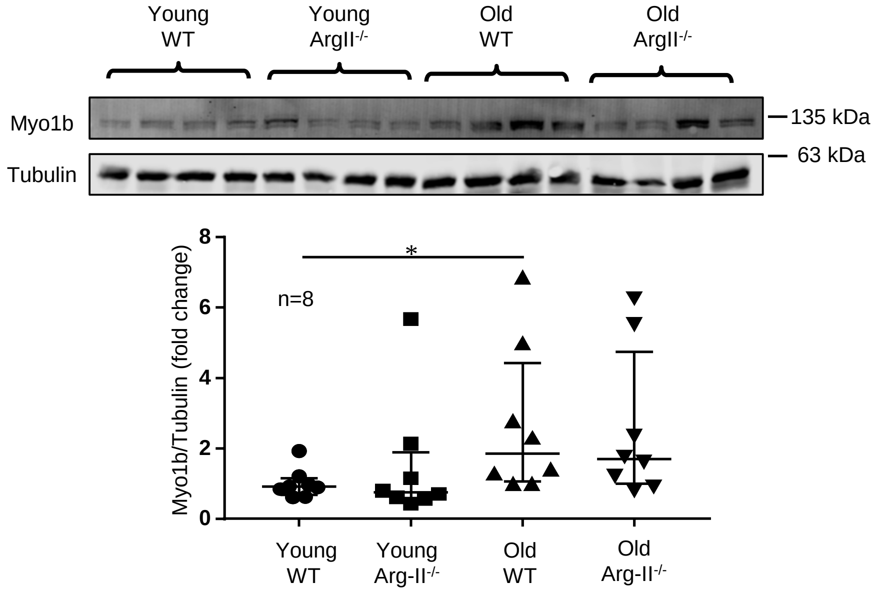

Fig. S6. Myosin 1b was elevated in aging mice aorta. Aortas isolated from young (5-7 months) and old (22-24 months) WT and $ArgII^{-/-}$mice were subjected to immunoblotting analysis of Myo1b and tubulin in female mice (**A,** n=10 mice in each group) and in male mice (**B,** n=8 mice in each group). *p<0.05 vs young wild type.
